# What Can We Learn from the Functional Clustering of Mortality Data? An Application to the Human Mortality Database

**DOI:** 10.1007/s10680-021-09588-y

**Published:** 2021-06-28

**Authors:** Ainhoa-Elena Léger, Stefano Mazzuco

**Affiliations:** Department of Statistical Sciences, University of Padua, Padua, Italy

**Keywords:** Functional data analysis, Clustering, Mortality, HMD

## Abstract

This study analyzed whether there are different patterns of mortality decline among low-mortality countries by identifying the role played by all the mortality components. We implemented a cluster analysis using a functional data analysis (FDA) approach, which allowed us to consider age-specific mortality rather than summary measures, as it analyses curves rather than scalar data. Combined with a functional principal component analysis, it can identify what part of the curves is responsible for assigning one country to a specific cluster. FDA clustering was applied to the data from 32 countries in the Human Mortality Database from 1960 to 2018 to provide a comprehensive understanding of their patterns of mortality. The results show that the evolution of developed countries followed the same pattern of stages (with different timings): (1) a reduction of infant mortality, (2) an increase of premature mortality and (3) a shift and compression of deaths. Some countries were following this scheme and recovering the gap with precursors; others did not show signs of recovery. Eastern European countries were still at Stage (2), and it was not clear if and when they will enter Stage 3. All the country differences related to the different timings with which countries underwent the stages, as identified by the clusters.

## Introduction

In recent decades, best-practice life expectancy has increased with unexpected rapidity and exceeded the highs previously held by several countries; laggards have been catching up, and former leaders have been falling behind (Oeppen & Vaupel, 2002). At the same time, interest is growing in health inequalities among countries, which are relatable to historical health crises as in Eastern European countries (Meslé et al., 2002) or long-standing health problems as in the United States (Shkolnikov et al., 2011). In most of the cases, the analysis of mortality trends is done by focusing on summary measures, such as life expectancy at birth $e_0$ or life disparity $e^{\dagger }_0$. For example, Amin and Steinmetz (2019) linked life expectancy with cardiovascular disease and cancer in individual states of the US by finding spatial clusters with higher values of $e_0$. Life expectancy at birth is also applied to evaluate the precision of mortality forecasts, even though Bohk-Ewald et al. (2017) has suggested that lifespan disparity could also be used. Lifespan disparity has also been advocated as a useful indicator to analyse the evolution of inequality in age-at-death across countries (Vaupel et al., 2011; Van Raalte et al., 2018). In other cases, scholars focus on specific components of mortality, disregarding the global pattern. For instance, Medford et al. (2019), analyzed lifespan after age 100 in Sweden and Denmark to show that the lifespans of Danish centenarians have been lengthening but not those of their Swedish counterparts. The shifting and compression dynamics of mortality at older ages have been extensively investigated (Kannisto, 2001; Canudas-Romo, 2008; Thatcher et al., 2010; Ebeling et al., 2018). As another example, Zanotto et al. (2020) focused their analysis on premature mortality. Therefore, it appears that analysing mortality evolution of one or more countries means choosing among a wide range of mortality indicators and focusing either on global mortality or a specific component. Meslé et al. (2002) already tried a clustering solution to group several European countries based on their age-specific death probabilities, highlighting clear differences in life expectancy trends and in the age structure between Eastern and Western countries that were more important than the traditional South-North division. More recently, Debón et al. (2017) grouped EU countries through fuzzy *c*-means cluster analysis of mortality surfaces and found similar results. Moreover, they raised the issue of the selection of mortality indicators to characterize the clusters and proposed the use of non-parametric techniques (e.g., classification and regression trees, or CART, and random forests) to rank indicators, based on their capacity to discriminate between-group inequalities.

Lee and Carter (1992) developed a model to forecast mortality based on the singular value decomposition of a matrix of the logged death rates by age and time, which identifies a single time-index of mortality changes and the mortality components or age-patterns. This model is the same that led Tuljapurkar et al. (2000) to suggest the existence of a universal pattern of mortality decline. Applying a cluster analysis on the Lee–Carter time-indices of many indicators and populations, Bergeron-Boucher et al. (2017) found similar patterns of mortality decline among non-Eastern-European countries but differences in the trends between females (linear trend) and males (accelerated trend). Many extensions of the Lee–Carter model have been proposed, including for multi-populations (Booth & Tickle, 2008) to obtain coherent forecasts (Li & Lee, 2005; Russolillo et al., 2011; Hyndman et al., 2013), as well as to deal with the limitation of assuming a constant rate of mortality improvement (Oeppen et al., 2008; Haberman & Renshaw, 2012; Li et al., 2013; Bohk-Ewald & Rau, 2017). Hatzopoulos and Haberman (2013) performed a fuzzy *c*-means cluster analysis based on the main time trends, which were estimated by means of a generalized linear model (GLM) model, to determine which countries had similar patterns and would be included in their coherent forecast model. The results divided the countries into different Eastern and Western clusters and support the idea of a single pattern of mortality decline across Western subpopulations. However, other works have suggested less homogeneity. For instance, McMichael et al. (2004) have show that there was an increased heterogeneity across countries, even though it should be noted that in their analysis both developed and poorer countries were considered.

In this work, we suggest the application of a functional data analysis (FDA) approach to mortality data, because it presents the advantage of considering smooth curves rather than scalar data. Such an approach (Ramsay & Silverman, 2005) is increasingly gaining ground among scholars and has become popular in demographic modeling and forecasting (Hyndman & Shang, 2009) and explanatory analysis (Hyndman & Shang, 2010). The aim of this paper is to explore the changes of age-specific mortality in low-mortality countries in the last few decades and to provide a comparative setting. More specifically, we propose a functional clustering of mortality profiles (e.g., in terms of age-specific rates), which can be seen as curves over age that can be observed for every country and every year. We suggest that taking a functional perspective can be an informative approach, as it allows the clustering of complete mortality profiles without losing sight of the role played by single components and reducing some of the inherent randomness in the observed data. The changes in mortality profiles will determine a country’s exclusion from or inclusion into a specific cluster at any time point, and in this way, we will be able to see whether the countries are evolving in the same way (i.e., following the same sequence of clusters) or different patterns are found. In addition, functional principal component analysis will be applied for the characterization of each group, providing a continuous setting for their interpretation and comparison.

The remainder of this paper is organized as follows. In Sect. 2, we explain our choice of mortality data from the Human Mortality Database (HMD) for 32 countries over the time range 1960–2018. In Sect. 3, we explicitly describe the functional representation of the mortality data and the advantages of working with a smoothed version. Next, FDA-based clustering techniques and the theory of functional principal component analysis are exposed in detail. In Sect. 4, the results are presented. We construct the smoothed version of the age-at-death distributions, and group the resulting curves using different methods of functional clustering to compare the mortality experience of the different countries separately for males and females. Only the results of one method are reported in the text, while the remaining ones can be found in the appendices. We then employ the functional principal component analysis to characterize the clusters. Finally, in Sect. 5, we discuss the results and offer some concluding comments.

## Data

We chose data from the Human Mortality Database (2020), which ensures a high quality and quantity of data on mortality profiles of many European and some non-European countries for a wide range of years. Of the 40 countries available, we excluded those with time series that were considered too short (Chile, Croatia, Greece, Israel, Slovenia, Korea, and Taiwan) and those with limited population sizes (Luxembourg and Iceland). As for the period, we chose to consider data from 1960 (after the Second World War and related economic crises) to 2018, which was the last year available for a majority of the countries. Considering that we needed to split the German data into East and West in order to examine it back to 1960, we had a final count of 32 countries.

**Fig. 1:**
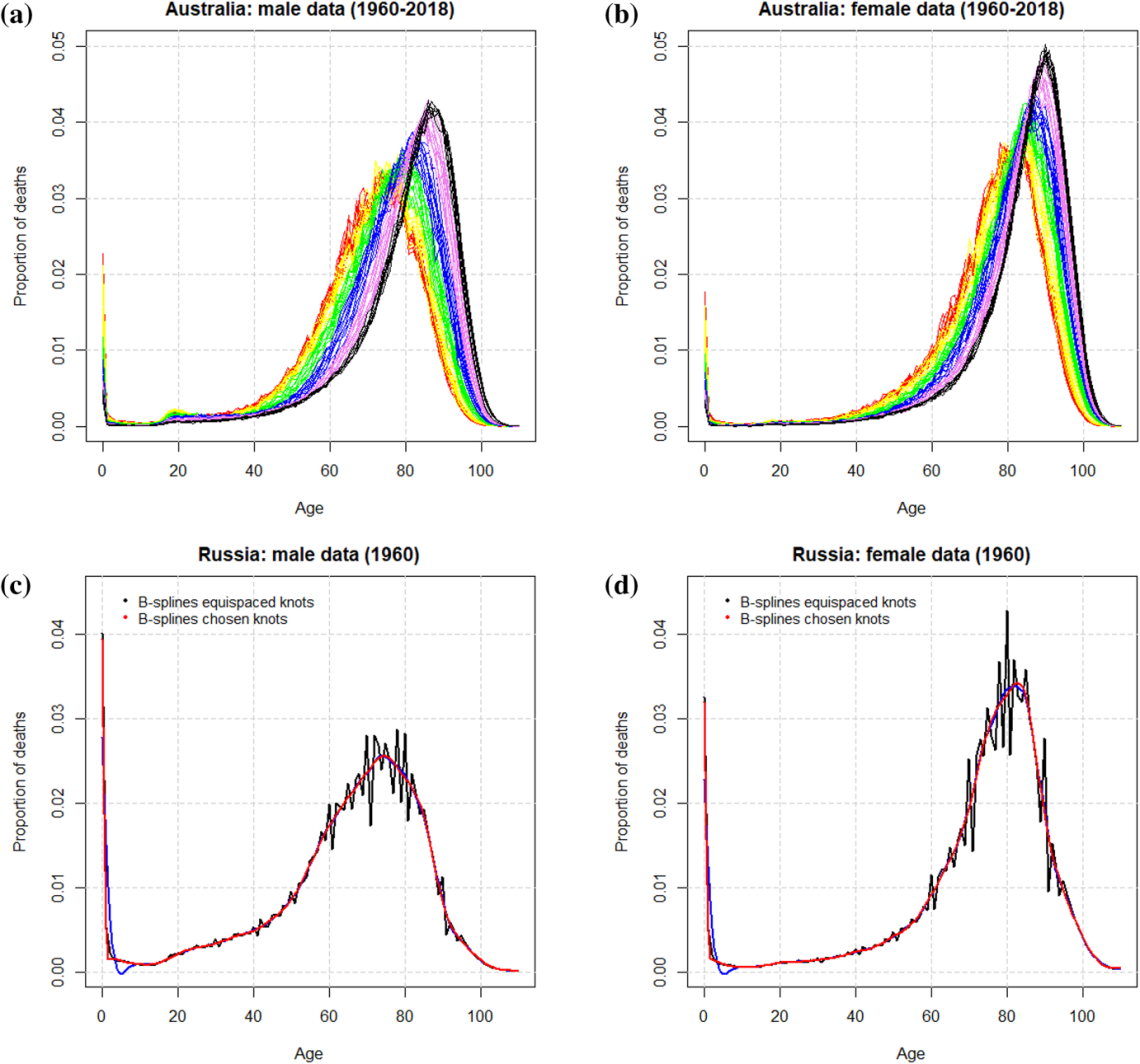
Plots of the life-table death count from 1960 to 2018 in a single-year group for **a** males and **b** females. Curves are ordered chronologically, the oldest years are shown in red and the most recent years in black. Smoothing of the curve for Russia in 1960 is shown with a sequence of 111 knots for **c** males and a sequence of 31 knots for **d** females, respectively. Every curve is smoothed with its specific $\lambda _{\mathrm{GCV}}$

We studied life-table death counts ($d_x$), where the life-table radix (i.e., a population experiencing 100,000 births annually) was fixed at 100,000 at age zero for each year. This means that for each combination of country and year, we had a curve of mortality age pattern for ages from zero to 110. Usually, age-specific rates are used for mortality analysis. However, we chose to use the age distribution of deaths because one of the most acknowledged transformations of mortality age patterns in developed countries over the past few decades has been the shift of the adult modal age at death (see, e.g., Canudas-Romo, 2008; Bergeron-Boucher et al., 2015; De Beer & Janssen, 2016) and the compression of deaths above the mode (Thatcher et al., 2010). These features are shown in Fig. 1 which presents mortality data for Australian males (a) and females (b) from 1960 to 2018; smoothed curves are also shown in (c) and (d), which are discussed later. The age distributions of death have been coloured according to the division of the period into six decades, in spectral order (red, yellow, green, blue and violet) and ending in black. More recently, Zanotto et al. (2020) have shown that premature mortality has also evolved in the last few years, with different patterns for several countries. All these transformations are better visualized from the age distribution of deaths ($d_x$) than from the age-specific rates ($m_x$). This explains why new models that fit the $d_x$ are emerging (Oeppen et al., 2008; Bergeron-Boucher et al., 2017; Mazzuco et al., 2018; Basellini & Camarda, 2019; Shang & Haberman, 2020). Moreover, mortality rates ($m_x$), survival probabilities ($l_x$) and the age distribution of deaths ($d_x$) are complementary mathematical functions, and each one can be derived from the others (Heuveline et al., 2001). This means they convey the same information; therefore, choosing one or another does not affect the results of the cluster analysis. However, as mentioned previously, using $d_x$ will allow a better visualisation of the transformations of the mortality profiles of the selected countries.

## Methods

Functional data analysis (FDA) refers to the statistical analysis of data that are in the form of functions and extends the classical multivariate methods. The monographs on functional data by Ramsay and Silverman (2005) develop the methodology and applications, and the book by Ferraty and Vieu (2006) on nonparametric models contains a review of the most recent contributions on this topic. In Sect. 3.1, we describe how to obtain a smoothed functional representation of the data. Section 3.2 introduces functional cluster analysis useful to group smoothed curves by country and year. Finally, Sect. 3.3 presents the theory of functional principal component analysis, which we employed to characterize and compare the clusters.

### Functional Data

Considering the data in a functional form means that we assume the existence of a continuous function giving rise to the observed data, so that a pair of adjacent data values are necessarily linked together to some extent and are unlikely to be too different from each other. Let $y(t_{1}),\dots ,y(t_{N})$ denote age-specific mortality data (i.e., mortality rates or life-table death counts) at ages ${t_{1},\dots ,t_{N}}$, which can be single years of age ($t_{1} = 0, t_{2} = 1, \dots$) but also 5-years-old age groups. A functional approach assumes that the discrete observations come from a continuous underlying function *x*(*t*) defined on $t \in [0,T]$. Formally, in the case of observations at the same instants on a common interval, functional data consists of a set of *n* curves denoted as $x_{i}(t_{j})$, with $t_{j} \in [0,T]$, $j=1,\dots ,N$, $i=1,2,\dots ,n$ and

1 $$\begin{aligned} y_{ij} = x_{i}(t_{j}) + \epsilon _{ij}, \end{aligned}$$

where the error term $\epsilon _{ij}$ contributes to the roughness of the raw data. The curves are assumed to be independent realisations drawn from the same continuous stochastic process *X*(*t*) belonging to $L_{2}[0,T]$ space. The first step in FDA is the reconstruction of the functional form from discrete data. To this aim, we will use a basis function system, which is a set of known functions that are independent on each other and that can arbitrarily approximate any function. Let us consider *p* known basis functions $\psi (t)=(\psi _{1}(t),\dots ,\psi _{p}(t))$. The basis function procedures represent the function *X*(*t*) by a linear expansion

2 $$\begin{aligned} X(t)=\sum _{j=1}^{p} \gamma _{j}\psi _{j}(t), \end{aligned}$$

where $\gamma =(\gamma _{1},\dots ,\gamma _{p})'$ are the basis function coefficients to be estimated by the ordinary least squares method minimising the sum of squared residuals. We use B-spline functions as they are the most common choice for non-periodic functional data. In practice, the interval over which the function is to be approximated is divided into *L* subintervals separated by values $\tau _{l}$, with $l=1,\dots ,L-1$, that are called knots. Over each subinterval, a spline is a polynomial of specified order *m*, and adjacent polynomials join up smoothly at the knots.

We usually want the underlying functions $x_{i}(t)$ to be smooth in order to capture the structural component of the data and reduce the noise of the data. There exist many possible approaches to control the irregularity of the curve and obtain a better approximation. Regression splines use the number of knots as a regulation parameter; the more knots used, the smoother the curve. In many applications, the knots are chosen to be equally spaced or are placed at the quantiles of the distribution. However, one can also place more knots in regions known to contain high curvature and fewer where there is less. More recently, adaptive knot selection procedures have been developed (Kaishev et al., 2016). In this work, we use smoothing splines, which introduce a roughness penalty term in the objective function. A natural measure of a function’s roughness is the integrated squared second derivative. Therefore, the penalised least square estimation criterion becomes

3 $$\begin{aligned} {\textit{PSSE}}_{\lambda }(x_{i}(t)|y))=\sum _{j=1}^{N} \big [ y_{ij}-x_{i}(t) \big ]^{2}+ \lambda \int \big [ D^{\prime \prime }(x_{i}(t)) \big ]^{2}{\mathrm{d}}t, \end{aligned}$$

where $x_{i}(t)=\sum _{j=1}^{p} \gamma _{ij} \psi _{j}(t)$ is the basis expansion of each curve, and $y_{ij}$ with $j=1,\dots ,N$ are discrete observations for the *i*th curve. The smoothing parameter $\lambda$ controls the trade-off between the closeness of fit to the average of the data and the variability of the curve and is commonly chosen subjectively or selected through the generalized cross-validation criterion. In our application, we chose to have the same set of knots for all the curves. Indeed, using different sets of knots would have affected the cluster analysis. In particular, inclusion to a specific cluster could depend on a different specification of knots. We use a limited sequence of knots, as suggested by Ramsay and Silverman (2005) for situations where a large number of sampling points is involved. Weights could also be included in Eq. (3) when it is not reasonable to assume that the measurement errors are independent or that they have the same variance. The weights should, ideally, be equal to the reciprocal of the variance-covariance matrix of the observations. We are not using weights, as we are applying the FDA to life table death counts. Thus, it is more reasonable to evaluate with equal weights given to each observation. Other smoothing methods have been developed in mortality analyses to improve forecasting. Hyndman and Ullah (2007) used penalized regression splines with a partial monotonic constraint to smooth the log mortality rates. Another widely used technique is the P-splines smoothing of Camarda et al. (2012), which combines (fixed knots) B-splines with a roughness penalty.

Once the functional representation of the data is obtained, we cluster the resulting smoothed curves through their basis expansions, and the functional principal component analysis will identify the major sources of variation in the data and help characterize the clusters.

### Functional Cluster Analysis

Cluster analysis is used to group countries by year for both sexes according to the dissimilarities among the smoothed curves. Age-specific mortality curves are divided into clusters so that they are as similar as possible within the same cluster and dissimilar as possible in different clusters. Because of the nature of the data itself (belonging to an infinite dimensional space), clustering functional data are generally a difficult task. Some common problem are the lack of definition for the probability density of a functional random variable, the definition of distances between curves and the estimation from noisy data. To overcome these problems, several methods have been developed that can be mainly grouped into three approaches (Jacques & Preda, 2014): two-stage clustering, non-parametric clustering (also called distance-based clustering) and model-based clustering.

A two-stage approach deals with the problem of the data dimension by first approximating the curves with a finite number of parameters (the filtering step) and then uses clustering algorithms for finite dimensional data (the clustering step). The filtering step can be performed either using the curves’ coefficients of the basis functions or by their first principal components, and classical clustering algorithms can then be used on them (in the next section, we explain the use of functional principal component analysis as a reduction technique). The first contribution to two-stage methods was from Abraham et al. (2003), in which *k-means* clustering is based on B-spline coefficients.

Distance-based methods for clustering generally consist of defining specific dissimilarities for functional data and then apply clustering algorithms with a hierarchical or *k-means* method. Indeed, considering distances when dealing with functional data can be too restrictive, and an alternative is to use a semimetric instead of a distance. Formally, a semimetric *d* in a functional space *F* is defined as an application on $F\times F$ that takes values in ${\mathbb {R}}_{+}$, such that autosimilarity, symmetry and the triangle inequality are fulfilled, but the identity property is not ($d(X_{i},X_{i'})=0 \not \Rightarrow X_{i}=X_{i'}, \forall (X_{i},X_{i'}) \in F \times F$). The families of semimetrics most widely used are based on derivatives and principal components (Ferraty & Vieu, 2006). In the latter case, the proximities between the two curves are computed while considering a truncated version of their basis expansion, obtained through principal components in a reduced dimensional space. If we consider the discretized curves $x_{i}$ and $x_{i'}$, the empirical version of the semimetric is

4 $$\begin{aligned} d_{q}^{FPCA}(x_{i},x_{i'})=\sqrt{\sum _{k=1}^{q} \bigg ( \sum _{j=1}^{J} (x_{i}(t_{j})-x_{i'}(t_{j})) [\phi _{k}]_{j} \bigg ) ^{2}}, \end{aligned}$$

with *q* as the number of principal components, and $\phi _{k}$ representing the eigenfunction of the covariance matrix associated with the eigenvalues $\lambda _{k}$ (a complete explanation of the FPCA procedure is found in the following section). This semimetric corresponds to the distance between the *q*-dimensional vectors of the principal component scores for the two curves. Therefore, the use of the semimetrics leads to a dimension reduction of the functional space, allowing the consideration of different curves in actuality as equal.

A model-based approach constructs homogeneous clusters by means of a density mixture model and allows the prediction of membership of each observation to one of the clusters. Conditional to the membership of a cluster, the observations are supposed to come from a common distribution with cluster-specific parameters. In the finite dimensional setting, the main tool to estimate the model is the multivariate probability density. In the case of functional data, the probability density is not defined, so we assume a density probability on the parameters describing the curves. The first model-based clustering method for functional data was developed by James and Sugar (2003).

Let $Z=(Z_{1},\dots ,Z_{K}) \in \{0,1\}^{K}$ be an unobserved random variable indicating the group membership of *x*(*t*): $Z_{k}$ is equal to 1 if *X* belongs to the $k^{th}$ group, and 0 otherwise. The clustering task aims therefore to predict the value $z_{i}=(z_{i1},\dots ,z_{iK})$ of *Z* for each observed curve $x_{i}(t)$. Each curve $x_{i}$ can be summarized by its basis expansion coefficient vector $\gamma _{i}$, as defined in Eq. (2), whose distribution is assumed to be a mixture of Gaussians with density

5 $$\begin{aligned} p(\gamma )=\sum _{k=1}^{K} \pi _{k} \phi (\gamma ;\mu _{k},\Sigma _{k}), \end{aligned}$$

where $\phi$ is the Gaussian density function and $\pi _{k}=P(Z_{k}=1)$ the prior probability of group k. Other distributions can be used, but in finite mixture models, Gaussian densities are by far the most commonly used, as they can reasonably approximate a wide class of probability distributions. This model is referred to as the functional latent mixture (FLM) model by Bouveyron and Jacques (2011) because it can be reparametrized to represent the curves through their group-specific eigenspace projection. The spectral decomposition of the matrix $\Sigma _{k}$ allows the modelling and interpretation of the variance of the data of the *k*th group through the parameters $a_{k1},\dots ,a_{kd_{k}}$ and the variance of the noise through parameters $b_{k}$, where $d_{k}$ can be considered as the intrinsic dimension of the latent subspace of the *k*th group, and $Q_{k}$ is the matrix containing the basis expansion coefficients of the eigenfunctions ($\text {FLM}_{[a_{kj}b_{k}Q_{k}d_{k}]}$). In contrast to the two-stage methods, in which the estimation of these parameters is done previous to clustering, the two tasks are performed simultaneously in this approach. The funHDDC algorithm (Bouveyron & Jacques, 2014) models and clusters the curves through their projections in the group-specific subspaces obtained by performing functional principal component analysis conditionally on the posterior probabilities of belonging to group *k*.

### Functional Principal Component Analysis

The principal component analysis is a statistical procedure mostly used for reducing the dimensionality of the data while losing as little information as possible. The use of principal component analysis to study mortality is not new and has been used with parameter estimation proposals in mortality forecasting (Lee & Carter, 1992; Booth et al., 2002; Renshaw & Haberman, 2006; Hyndman & Ullah, 2007). Functional principal component analysis (FPCA) is the extension of the more classical multivariate PCA to functional data. In our work, we use FPCA for clustering purposes but also for data projection and the interpretation of the curves.

As in the multivariate case, FPCA provides a way of looking at covariance structure that can be much more informative and can complement a direct examination of the variance-covariance function. The values of the variables in PCA are replaced by function values $x_{i}(t)$ in FPCA and the discrete index by the continuous index *t*. Given *n* functional observations $x_{i}(t)$ with $1\le i \le n$ and ${\bar{x}}(t)$ as the estimate of the mean function, the estimated covariance function, analogous with the covariance matrix in the multivariate case, is defined as:

6 $$\begin{aligned} S(s,t) = \frac{1}{n-1} \sum _{i=1}^{n} (x_{i}(s)-{\bar{x}}(s))(x_{i}(t)-{\bar{x}}(t)). \end{aligned}$$

The spectral decomposition performs the task of finding the most important modes of variation in the covariance or correlation matrix of the curves. It provides a countable set of positive eigenvalues $\lambda _{1} \ge \lambda _{2} \ge \cdots$ associated with a basis expansion of orthonormal basis functions $\phi _{l}(t)$ with $l=1,\dots$ such that

7 $$\begin{aligned} S(s,t) = \sum _{l=1}^{\infty } \lambda _{l} \phi _{l}(s) \phi _{l}(t). \end{aligned}$$

In standard terminology, the basis functions $\phi _{l}(t)$ are the eigenfunctions or harmonics; they define the most important modes of variation in the curves and are orthogonal of each other. The eigenvalues measure the variability in the directions corresponding to the eigenfunctions.

The projection of $x_{i}(t)$ in the direction of the eigenfunctions $\phi _{l}(t)$ provides us with the functional principal components, a set of zero-mean linearly uncorrelated random variables, defined on the same interval of the functional data, with variance $\lambda _{l}$. As $x_{i}(t)$ and $\phi _{l}(t)$ are functions, summations of variables in the multivariate context are replaced by integrations over *t* to define an inner product. Thus, the principal component scores of the *i*th curve are defined as

8 $$\begin{aligned} c_{i,l} = \int x_{i}(t) \phi _{l}(t) {\mathrm{d}}t. \end{aligned}$$

The decomposition of Karhunen-Loève allows the expression of the curve through its functional principal component expansion

9 $$\begin{aligned} x_{i}(t) = \sum _{l=1}^{\infty } c_{i,l} \phi _{l}(t). \end{aligned}$$

Therefore, the FPCA provides us with a group of basis functions $\phi _{1}(t), \dots , \phi _{l}(t)$ and returns functional data as a linear combination of the new basis functions, where the coefficient of the $\phi _{l}(t)$ is the estimated score of the l-th principal component of the corresponding curve. The decomposition of Karhunen-Loève facilitates the dimension reduction in that if the first *q* terms (for a large enough *q*) provide a good approximation to the infinite sum, the information contained in the curve $x_{i}(t)$ is essentially synthesized by the *q*-dimensional vector $c=(c_{i1},\dots ,c_{iq})$, and one can work with this approximation.

FPCA is useful for the dimension reduction of the curves in all the clustering approaches applied to the low-mortality countries. In addition, the eigenfunctions allow the identification of the main directions of variability in the complete mortality profile with respect to the mean curve, and the corresponding scores for every curve can be used to characterize the countries in the clusters in a reduced dimensional space.

## Results

### From Discrete Data to Smooth Curves

Although in functional analysis there is no general requirement for the data to be smooth, we find in some cases particularly noisy data makes smoothing necessary. In the current study, this problem affected most of the curves of the Eastern countries at the beginning of the period and was attributable to the quality of the data. We thus used a basis expansion of B-splines to obtain a smoothed representation of the data by means of the **R** package fda (Ramsay et al., 2011). We chose to use the roughness penalty method described in Sect. 3.1 because it allows continuous control over the smoothness. We employed the same set of knots for every curve so that the estimation of the splines coefficients was performed on the same age intervals. This is more appropriate for the functional cluster analysis and FPCA that will be applied in the following on the basis coefficients. In order to maintain the data structure, two sequences of knots over the age range [0, 110] have been evaluated: a sequence of 111 equally distributed knots (i.e., one for every age); and a sequence of 31 knots, one every three months over the age interval [0, 2] and one every 5 years over the age interval [2, 110]. The latter has been preferred to the former, not only as it is more parsimonious, but also because it is preferable in terms of the goodness of fit. As an example, as shown in Fig. 1, both solutions of knot sequences were applied to the curves of Russian males (c) and females (d) in 1960. The comparison reveals that 31 knots unequally distributed better followed the steep decrease of infant mortality in the first two years and respected the unicity of the mode distribution. In this example, the smoothing parameters were selected through the generalized cross-validation (GCV) criterion. GCV is a mean-squared error based measure, twice discounted by a term taking into account the number of parameters and the magnitude of the smoothing parameter. In the following analyses, two alternatives for the smoothing parameter have been applied to the curves: a common smoothing parameter ($\lambda _{\mathrm{GCV}}^{\mathrm{COM}}=0.0025$) and a different smoothing parameter for each curve. As the results of the two alternatives did not show any relevant differences, we will present only the ones obtained with a curve-specific $\lambda$.

### Analysing Mortality Evolutions Through Functional Clustering

The analyses of this section focus on the classification of mortality curves to understand the patterns or trajectories for the selected period and developed countries. Functional analyses were performed separately for males and females, due to the fact that in the past, these populations experienced different mortality trends. The three methods of functional clustering described in Sect. 3 have been carried out: two-stage on the coefficients of basis expansion of the curves, model-based with the FLM model and distance-based through a semimetric using FPCA. The model-based method was performed with the package funHDDC (Bouveyron & Jacques, 2014), whereas for the distance-based approach through a semimetric, we used the package fda.usc (Febrero Bande & Oviedo de la Fuente, 2012), which extends the functionalities of the fda package.^1^ We will show the model-based method for men and the distance-based method for women, while the other methods are presented in the appendices. This choice stems from the different patterns shown by the men and women mortality cluster solutions and are explained in the following.

For the model-based clustering with the men’s data, we chose the reduced model $\text {FLM}[a_{kj}b_{}Q_{k}d_{k}]$ with a common *b* parameter for groups as the observations were obtained in the same data acquisition process, and it was natural to assume a common behavior of the noise outside the latent subspaces. The number of clusters was selected according to the Bayesian information criterion (BIC) defined with a positive log-likelihood and to model the complexity (i.e., the number of parameters). As a local maximum occurred at $K=5$ and the increase in model complexity is greater after $K=5$ (Table 1), we chose the partition with five clusters. The trend of BIC values as well as the stability of cluster dimensions was verified by initializing the classes of the funHDDC algorithm with the *k-means* function and setting different seeds.

**Table 1:** Model-based clustering for men: the BIC values and model complexity (${\text {set.seed}}=5555$) for the choice of the number of clusters

No. of Clusters	BIC	Complexity
$K=2$	− 2,348,123.08	229
$K=3$	− 1,294,815.85	328
$K=4$	− 277,431.79	395
$K=5$	− 22,057.17	493
$K=6$	− 243,277.24	656
$K=7$	− 183,021.71	723
$K=8$	− 79,862.68	851

**Fig. 2:**
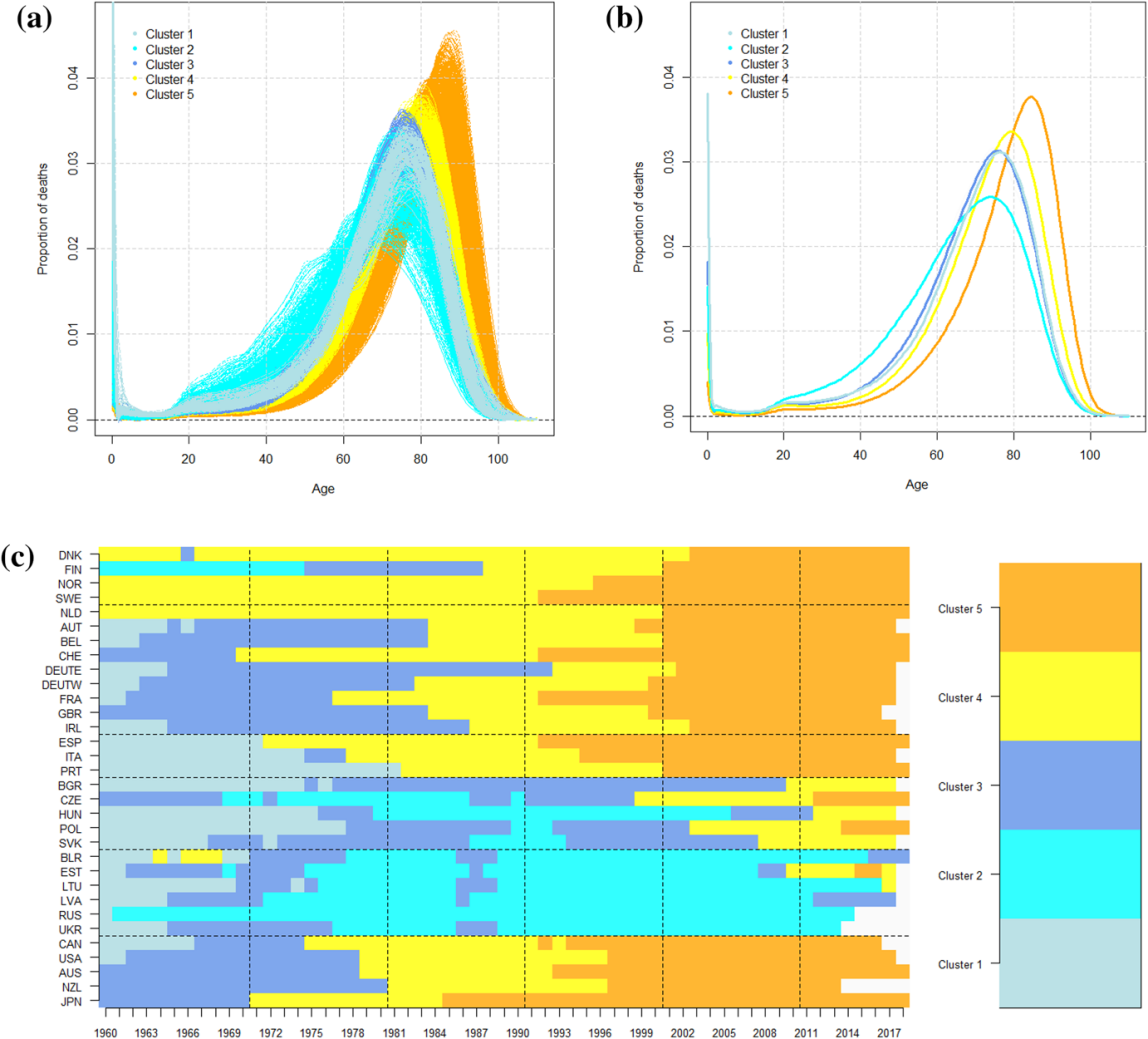
Results of the model-based clustering on the men’s mortality data: **a** mortality curves, **b** mean curves and **c** the composition of the five clusters (9.26%, 16.26%, 23.42%, 26.17%, and 24.88% of the units)

The mortality curves and corresponding mean curves within the clusters, Fig. 2a, b, allow one to distinguish very clearly those with a similar shape but different levels of infant mortality and those with a higher accidental and premature mortality. Cluster 1 contains the curves with high infant mortality (4% on average), and Cluster 3 the ones with a similar shape but lower infant mortality (2% on average). Cluster 2 expresses a high level of premature mortality and a lower number of deaths around the modal age at death compared to other clusters. The shift toward older ages and the compression above the modal age at death is also clearly visible. Clusters 4 and 5 show a gradual shift, and the number of deaths increases around the modal age at death. Figure 2c shows how mortality curves were classified in the clusters and allows one to follow the evolution of countries (rows) from 1960 to 2018 (columns). The Northern, Western, Southern and extra-European countries experienced a decrease in infant mortality, followed by a shift of the curves and an increase in the number of deaths around the modal age at death over the whole period. The Nordic countries—which are well-known precursors of epidemiological transition—were already at Cluster 4 at the beginning of the period. Finland is an exception to this rule, but this comes as no surprise; this is a peculiar Finnish pattern of mortality (with an extremely high incidence of external causes of death) that has been already observed (Saarela & Finnäs, 2008). It should also be noted that Finland and Denmark joined the last cluster much later than Sweden and Norway. The Netherlands had a pattern similar to the Nordic countries, while Switzerland, France, Japan and Western Germany were a bit behind at the beginning (Cluster 2) but had a faster transition to Cluster 5. The Southern European countries (Italy, Spain and Portugal) started even farther back (Cluster 1) but also underwent a rapid transformation, which brought Italy and Spain to Cluster 5 at the same time as Sweden and Norway. Such a transition was slower in the United Kingdom, Ireland, Portugal and East Germany. The analyses also identified the higher infant mortality of Southern countries in the first twenty years (Cluster 1). In the second half of the period, the disparities seemed to be reduced, and all the countries followed the shifting and compression process of the mortality curves previously described (all ending in Cluster 5). Also, the Central countries reduced their high infant mortality in the first decade of the period (Clusters 1 and 3), but then had a delay of about 20 years with respect to the previous countries (reaching Cluster 4 only in the 2000s). The Czech Republic and Poland seemed to benefit from a more favourable situation, while Bulgaria, Hungary and Slovakia showed a slight shift and compression of curves only in the last decade of the period. Hungary was also characterized by a long period of increased premature mortality (belonging to Cluster 2 in the 1980s and 1990s). The former USSR countries were (not unexpectedly) those with the highest delay in the transition, and some of them were still stuck in Cluster 2, suggesting high levels of premature mortality.

A hierarchical cluster analysis was performed on the women’s data according to a distance-based approach with a semimetric using the functional principal components. Our decision to keep the first six components was due to the necessity of an approximation of the curves that accounted for all the components of mortality (for more details, see Appendix 1). The partition in five clusters was the more parsimonious, allowing one to distinguish the decrease in infant mortality, the shift of the curves to the right and the increase in the number of deaths around the modal age at death.

**Fig. 3:**
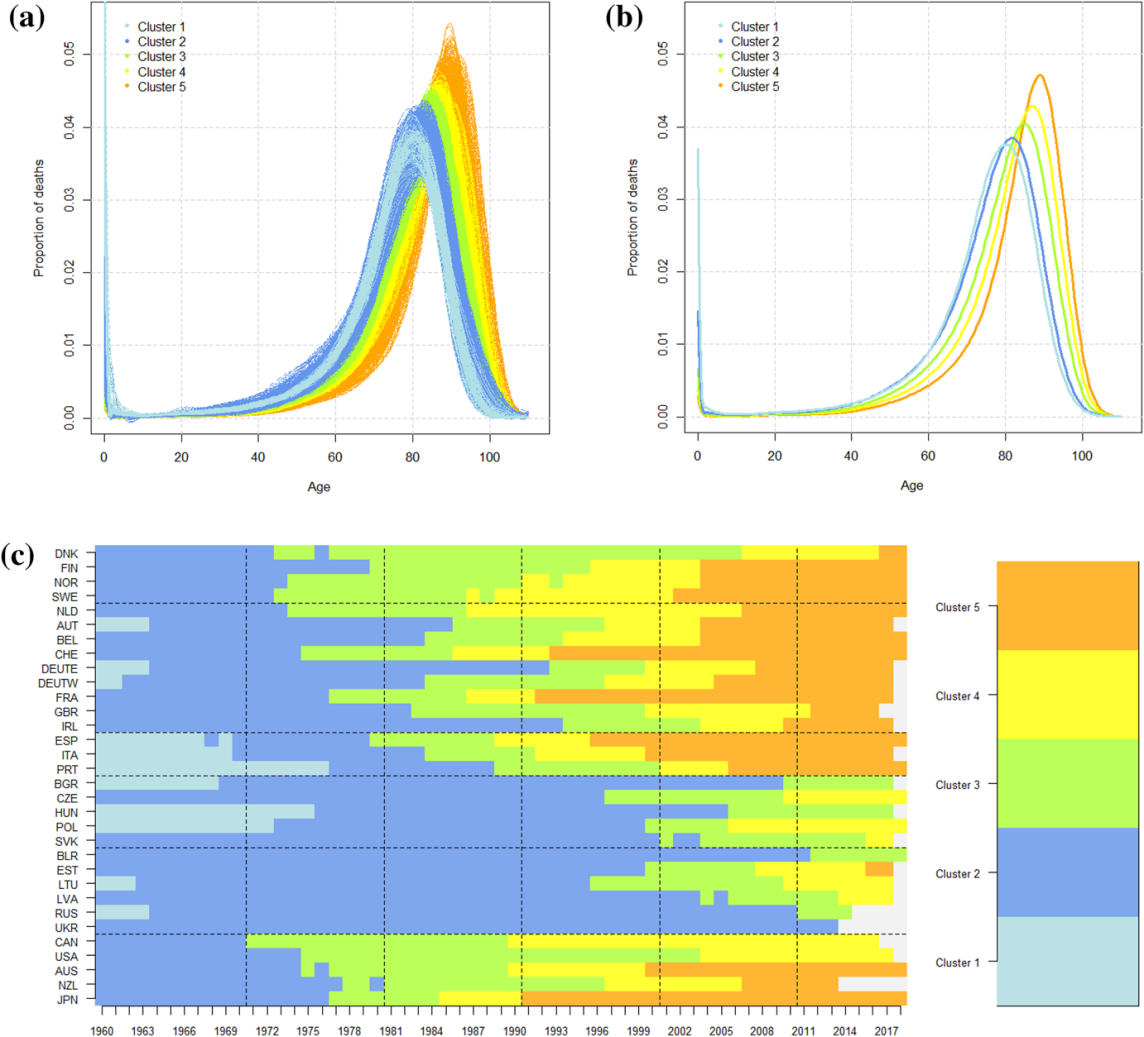
Results of distance-based clustering on the women’s mortality data: **a** mortality curves, **b** mean curves and **c** the composition of the five clusters (4.90%, 44.43%, 21.27%, 13.89% and 15.51% of the units)

As we can see from Fig. 3, Cluster 1 contains the curves with high infant mortality (4% on average); in Cluster 2, the curves have the same shape but lower infant mortality (2% on average). Clusters 3, 4 and 5 identify the curves characterized by a shift to the right and a compression around the modal age at death. Over the period, the Northern, Western, Southern and extra-European countries experienced a continuous shift and compression of the curves of mortality toward the older ages (Clusters 3, 4 and 5). However, disparities across the countries seemed to persist until the end of the period, as the transition to the clusters occurred in different years. For instance, Norway, Sweden, Switzerland, France, Spain and Japan anticipated the shifting process in the 1970s (Cluster 3), during the 1980s (Cluster 4) and at the beginning of the 1990s (Cluster 5). Some sex-specific dynamics can also be noticed like the stagnation of Denmark between the 1980s and 1990s that was attributable to a worsening of health conditions linked to smoking behavior (Lindahl-Jacobsen et al., 2016). Indeed, Denmark lagged far behind in the second part of the period and was the last country passing to Cluster 5 in 2004. Concerning Central and Eastern Europe, we can observe a long stationary period (Cluster 2) followed by a shift and compression of the curves during the last decade (Clusters 3 and 4). The Czech Republic, Poland and the Baltic countries seemed to be slightly advanced (ending in Cluster 4).

To sum up, the analyses for the data on both the men and women showed a similar evolution for the Northern, Western, Southern and extra-European countries that was characterized by the shift of curves to older ages and by the concentration of adult mortality around the modal age at death. For these four areas, we can thereby conclude the existence of a common pattern of evolution. In the case of the men’s data, all the countries belonged to the same group at the end of the period, supporting the hypothesis of an increasing homogeneity. The situation was more heterogeneous for the Central and Eastern countries because they did not experience the same evolution and, at the end of the period, they did not arrive at the same cluster. The comparison of the analyses of the men’s and women’s data revealed two different scenarios for the Eastern countries, characterizing the increase of premature mortality after 1990 as an entirely male phenomenon. The other methods of clustering show similar evolutions based on the same components of mortality and are presented in Appendix 2.

### Focus with Functional Principal Components Analysis

FPCA represents a useful tool for synthesising the variability of data and visualising the curves in a reduced dimensional space. Thanks to this technique, we were able to highlight the different features of the clusters and to interpret the associated patterns for some selected countries. In order to interpret the eigenfunctions (harmonics), we will represent the variation around the mean, which is a typical representation in FDA (Ramsay & Silverman, 2005). Moreover, the principal subspace faciltates the comparison by plotting for each individual the scores of the two principal components for some representative countries.

**Fig. 4:**
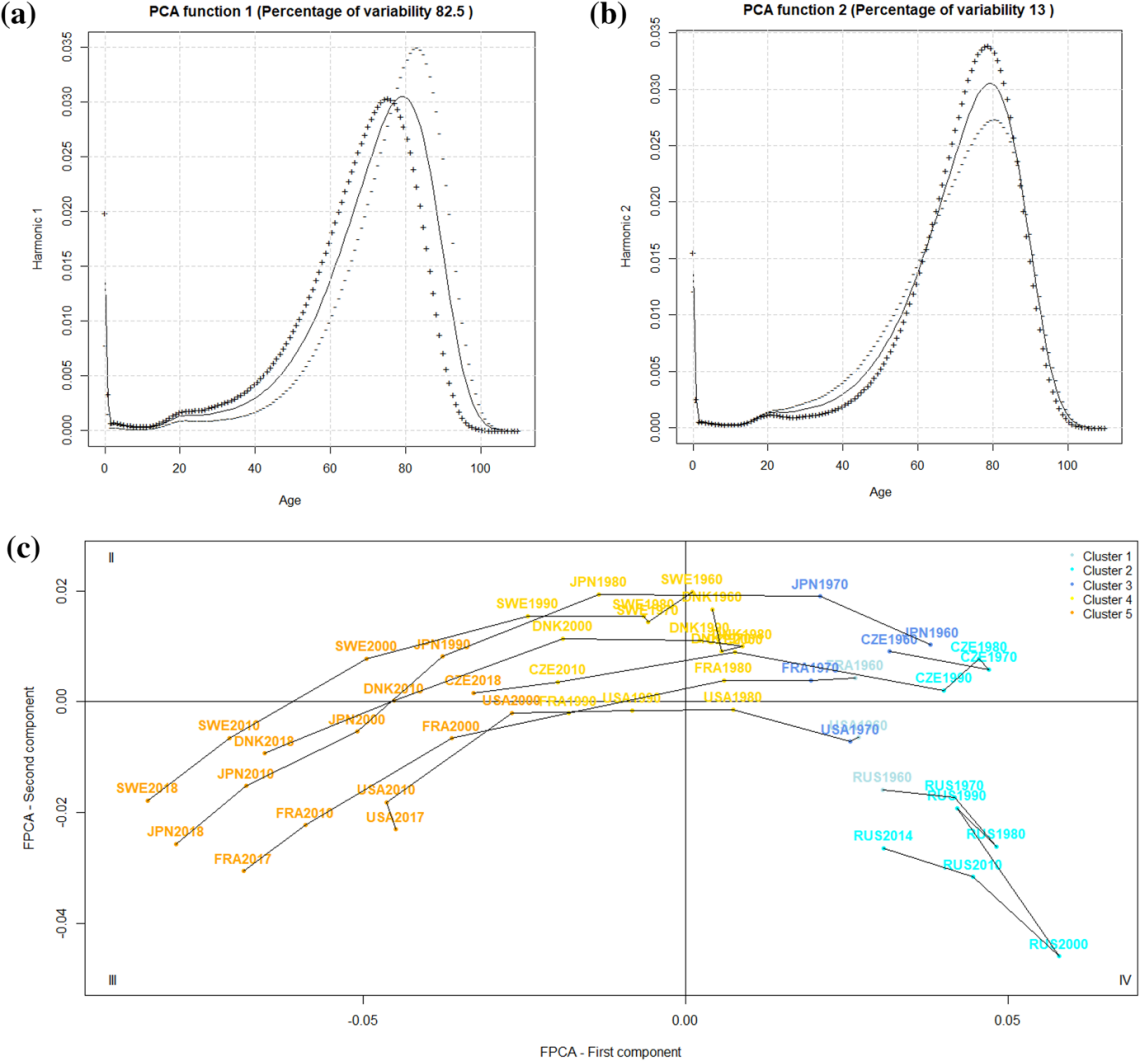
Results of the FPCA on the men’s mortality data: **a**, **b** group means and effect of the components and **c** first principal subspace with selected countries. The colours indicate the group memberships provided by the model-based clustering

From the FPCA for the men’s data, it emerged that most of the variability was explained by the first two principal components (82% for the first principal component and 13% for the second principal component). Figure 4a, b show a solid curve for each of the first two principal components, which is the overall smoothed mean for the men and the functions obtained by adding (+) to and subtracting (−) from the mean function an appropriate multiple of the eigenfunctions, ${\bar{X}}(t)\pm 2 \cdot \sqrt{\lambda _{i}}\phi _{i}(t)$), with the $\lambda _{i}$ eigenvalue of the *i*th component. Thus, the (+)/(−) curves represent the variation around the mean. Looking at Fig. 4a, we can see that the first eigenfunction has the effect of shifting and compressing the overall mean over the entire age range, because adding the first eigenfunction to the mean shifts the (+) curve to the left, and subtracting the first eigenfunction from the mean shifts it to the right and compresses the (−) curve. The curve of a country-year with a large negative score of the first principal component behaves more similarly to the (−) curve, while the curve of a country-year with a large positive score of the first principal component behaves more similarly to the (+) curve. Looking at Fig. 4b, we can see that the second eigenfunction has the effect to shape premature mortality (ages 20–65) and adult mortality (ages 65–85), because adding the second eigenfunction to the mean reduces the premature mortality and increases adult mortality, and subtracting the second eigenfunction from the mean increases the premature mortality and reduces the adult mortality. Again, a large negative/positive score of the second principal component makes the curve of a country-year behave similarly to the (−)/(+) curve. The fact that premature mortality and adult mortality are opposed is apparent as the (−) and (+) curves cross at approximately 65 years and move in opposite directions with respect to the mean curve. As we are dealing with a distribution, deaths occurring at younger ages avert deaths at older ages. Therefore, we can summarize that the first component is representative of the shift and compression of death distributions observed in the latest decades, while the second component is related to premature mortality. This is an interesting result, as it confirms that the shift and compression of mortality schedules are intertwined (Bergeron-Boucher et al., 2015), that the premature mortality component is independent of the shift and compression and 13% of the variability in the men’s mortality schedules are attributable to it.

Now that the interpretation of the components is clear, the mapping of the countries on the first two components will allow the description and comparison of the curves on the basis of the phenomena of shift, compression and amount of premature mortality. In Fig. 4c, the scores of the curves on the two first principal components are illustrated for seven representative countries (Denmark, Sweden, Japan, France, the Czech Republic, the United States and Russia) and coloured based on the membership to the five clusters obtained from the model-based clustering. We selected time intervals of 10 years for ease of interpretation (see Appendix 3 for the plot of all the considered years). The first principal subspace shows similar trajectories on the first component for Denmark, Sweden, Japan, France, the Czech Republic and the United States. Indeed, the decrease of the scores from positive to negative values discriminates these countries throughout the whole period and reflects the gradual shift and compression of mortality curves with respect to the mean curve, see Fig. 4a. Sweden was the only country that was always characterized by negative values, indicating behavior near the (−) curve and thus, an above-average shift and compression already at the beginning of the period. On the other hand, the Czech Republic started from the highest positive values of the first component and had a huge delay in the shift and compression, reaching the mean curve (vertical axis) only in 2000. In 2010, the Czech Republic was comparable to Sweden in 1990, equivalent to a delay of 20 years. Despite the general shift and compression (except from Russia), all the countries presented different levels of the second principal component. Sweden can again be seen as a reference country, as it was characterized by the highest values and, thus, by the lowest premature mortality, whereas the United States presented the lowest values and was, thus, characterized by more extended curves. Only Russia remained for the whole period in quadrant IV and experienced a completely different trajectory. The scores of the first and second principal component evolved in the opposite direction compared to the other countries revealing a shift to the left and an expansion of the curves. The lowest point was reached in 2000, after which the trend reversed.

**Fig. 5:**
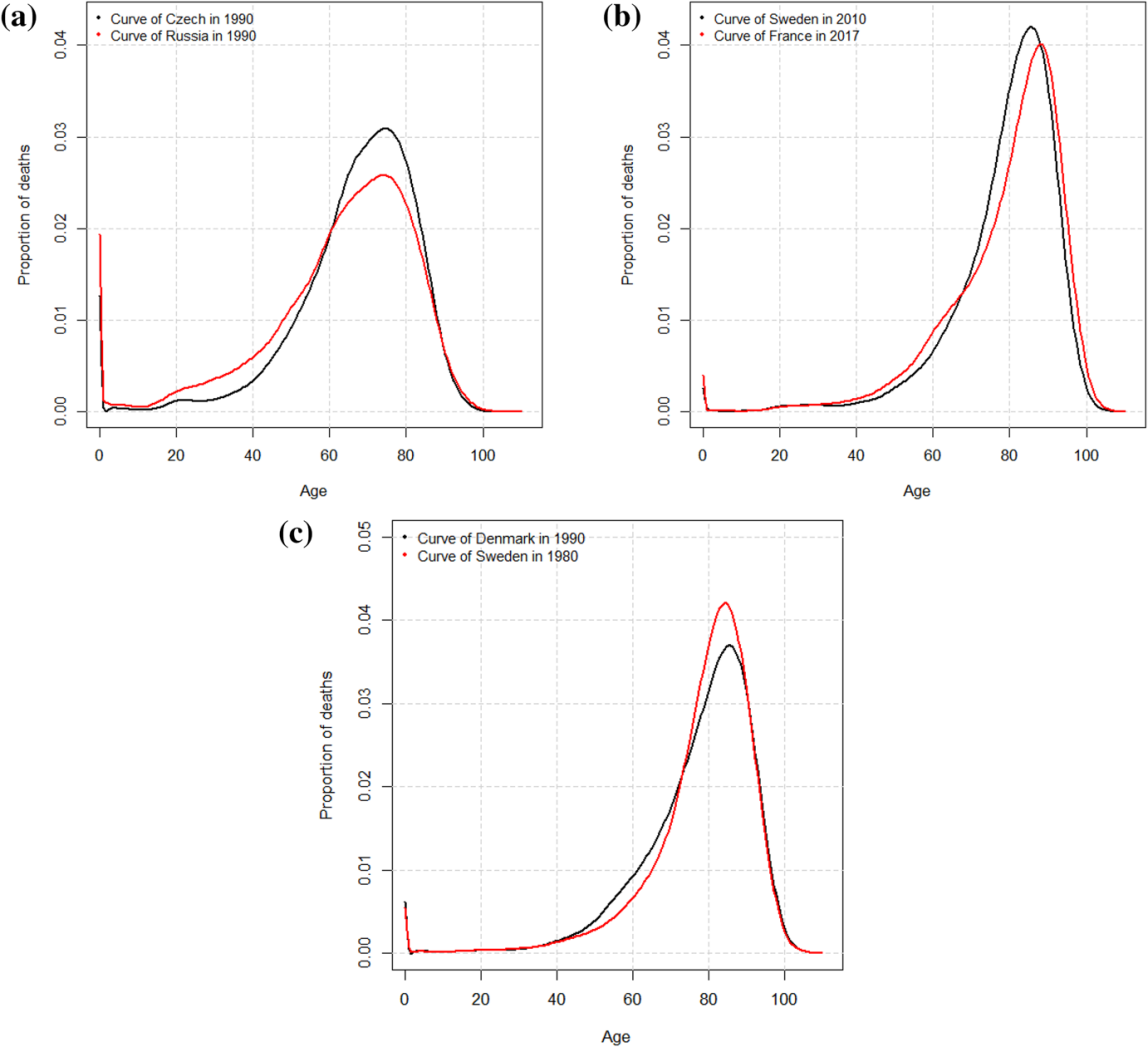
Comparison of smoothed curves in the same cluster: **a** men, the Czech Republic and Russia in Cluster 2; **b** men, Sweden and France in Cluster 5; and **c** women, Denmark and Sweden in Cluster 3

Therefore, the FPCA confirms the results of the previous cluster analyses and brings the advantage of characterizing the clusters with both components simultaneously. For example, at the beginning of the period, the Czech Republic was classified in the same cluster as Russia because of the positive large score on the first component, although it was very similar to the Western countries on the second component, see Fig. 4c. Figure 5a represents the curves of the Czech Republic and Russia in 1990 showing their similar position but different levels of premature mortality. Likewise, the comparison of France and Sweden at the end of the period (Cluster 5) reveals the same shift and compression but a higher premature mortality before the age of 65 in France, see Fig. 5b. In this respect, Zanotto et al. (2020) hypothesised that the behavior on the left slope was not attributable to an increase in the incidence of some causes of deaths but rather the strong shift and compression could have isolated premature mortality.

**Fig. 6:**
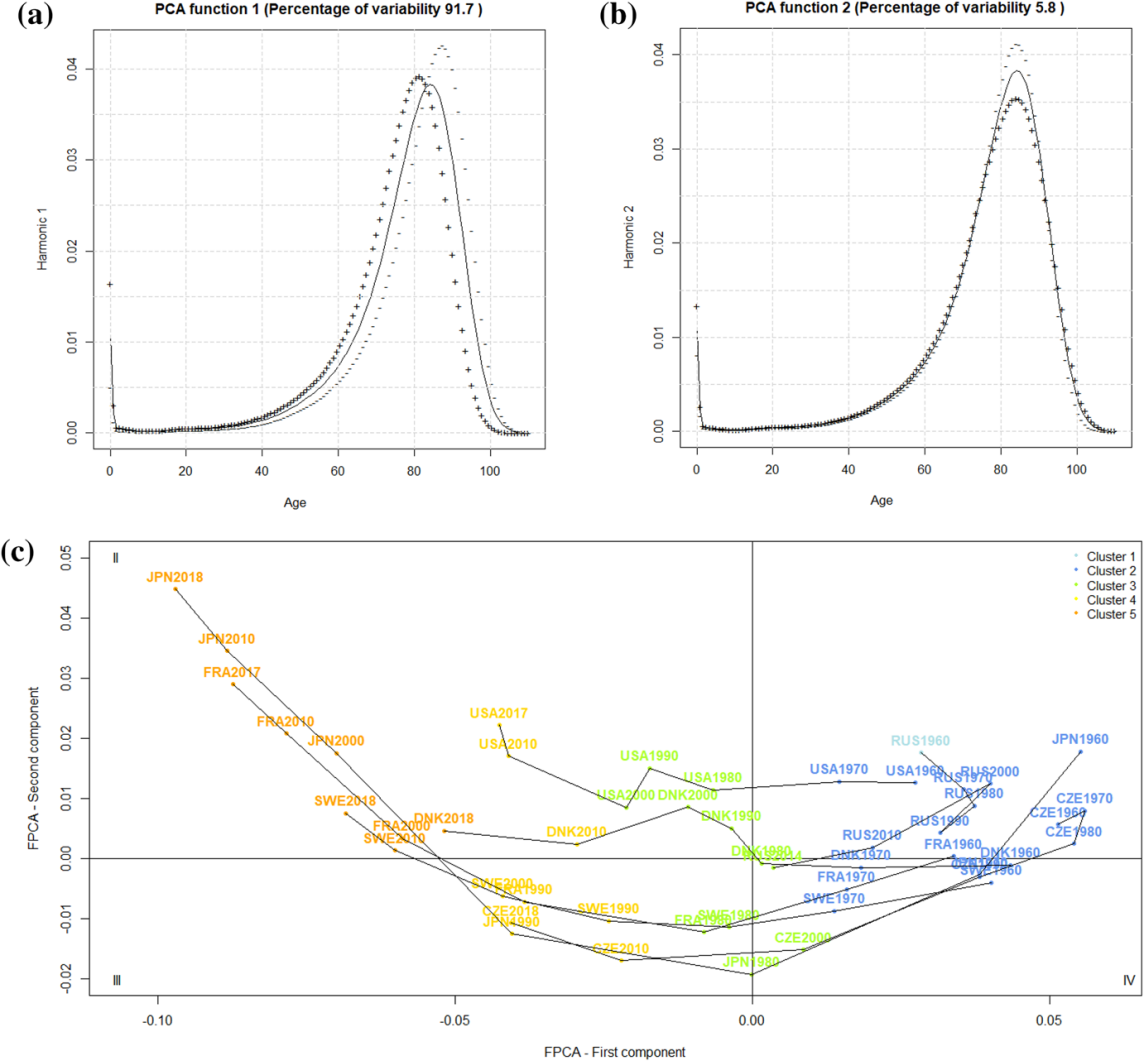
Results of the FPCA on the women’s mortality data: **a**, **b** group means and the effect of the components and **c** the first principal subspace with selected countries. The colours indicate the group memberships provided by the distance-based clustering

Concerning the women’s data, the FPCA revealed that most of the variability was explained by the first component (92%), with the second principal component being less relevant (6%). Figure 6a shows that the first eigenfunction has the effect of shifting and compressing the mean function from age 40 throughout adulthood and senescence, because adding the first eigenfunction to the mean shifts the (+) curve to the left, and subtracting the first eigenfunction from the mean shifts it to the right and compresses the (−) curve. A large negative/positive score of the second principal component makes the curve of a country-year behave similarly to the (−)/(+) curve. The effect of the second eigenfunction (b) is not so straightforward and seems to be a proxy of the compression around the modal age at death. Indeed, adding the second eigenfunction to the mean reduces the number of deaths around the modal age at death, while subtracting the second eigenfunction from the mean increases the number of deaths around the modal age at death.

On the first principal subspace, Fig. 6c, the first axis expresses the shift and compression of the curves and discriminates the countries over the period. All the countries experienced the shift and compression of the curves toward older ages although with different timings. The decrease of the scores was more pronounced for Japan, which started from large positive values and reached the largest negative values of the first component at the end of the period. As all countries had data up to 2010, it can be said that, in this year, the curves of France and Japan were the most shifted and compressed at older ages. Regarding the Central and Eastern countries, the Czech Republic stagnated until 1990 but then seemed to follow the pattern already described. Russia showed a slight improvement from 2010 and reached the mean curve (vertical axis) in 2014.

Once again, the FPCA allowed the characterisation of the clusters, and we will focus on the trends in Cluster 3. The stagnation of Denmark (Lindahl-Jacobsen et al., 2016) appeared from the higher scores on the second principal component between the 1980s and 2000 compared to other countries. Indeed, the curves of Sweden and Denmark with the same shift and compression reveal that the lower number of deaths around the modal age was linked to the increase in premature mortality in Denmark, see Fig. 5c.

## Concluding Remarks

In this study, we inspected the evolution of mortality schedules in HMD countries by means of a functional clustering method, which allowed us to consider mortality patterns as functions and avoid analysing only a component of mortality (e.g., infant or old-age mortality) or a summary measure like life expectancy, which is a mixture of all mortality components but without a clear distinction of their contribution to longevity progresses.

Three different methods of functional clustering have been considered: a two-stage method based on spline coefficients, a distance-based method through principal components (FPCA) and a model-based one. The latter method seemed to better reflect the men’s mortality evolutions in terms of changes in the real data, but the FPCA was also useful in determining what were the most relevant components that drove the transformations we observed in the last sixty years in HMD countries. The results showed that the two components accounted for 95% of the variability in the men’s mortality schedules: 82% for a component that can be explained in terms of the shift and compression of mortality and 13% for a second component that accounts for premature mortality. This demonstrates that shift and compression processes were mutually dependent, while premature mortality was an additional independent component, which accounted for a much lower (13%) but not irrelevant share of variability.

The results from the clustering provided us with many insights, although none of them came as a surprise. First, the results confirmed that homogenisation was taking place among most of the considered countries, as many of them followed the same evolution through the clusters. However, the men’s and women’s patterns were quite different, because for the men, most of the countries were included in the same cluster in the latest years, and countries from Eastern Europe not only lagged behind with respect to Cluster 5 but also did not show signs of a recovering process. The women’s situation was a bit different because of the homogeneity of the Northern, Western and Southern European countries, and the extra-European ones were less pronounced (Denmark, the United Kingdom, the United States and East Germany did not reach the highest longevity cluster). However, the Central and Eastern European countries looked much closer like their precursors. The difference between the men’s and women’s data was also characterized by the higher importance that premature mortality had for the former. Therefore, if the longevity of Eastern European men was still stagnating, that is partly attributable to the premature mortality, which is notably high in that area. The results also clearly show the stagnation periods that Denmark and the United States underwent at different times, which was much more visible in the women’s data. This stagnation prevented these two countries from joining the highest longevity group. Considering the latest evolution of United States longevity (Woolf & Schoomaker, 2019), the lag is going to persist (or even increase) for this country, while Denmark seems to be catching up, as can also be seen from Fig. 6.

This work, however, was also meant to show the potential of functional data analysis demographic studies, in which the leading forces of population growth (fertility, mortality and migration) are often measured in terms of age-specific rates or probabilities that reveal several components. Thus, similar analyses can be implemented on fertility and migration age patterns. Moreover, FDA allows other kinds of analyses: regression (both on scalar and functional covariates) and hypothesis testing. Therefore, we advocate an increasing implementation of such an approach to population studies. For example, we suggest that a further evolution of this work could consider the time dependence of curves in the same countries. Here, every year has been considered independently, and the time evolution of countries has been analyzed by inspecting when each country had moved from one cluster to another. Another approach could be that of combining a time-series approach (for instance, by means of a vector autoregressive model) with the functional approach presented here. In this way, countries can be clustered in terms of the evolution of their curves.

## Figures and Tables

**Fig. 7:**
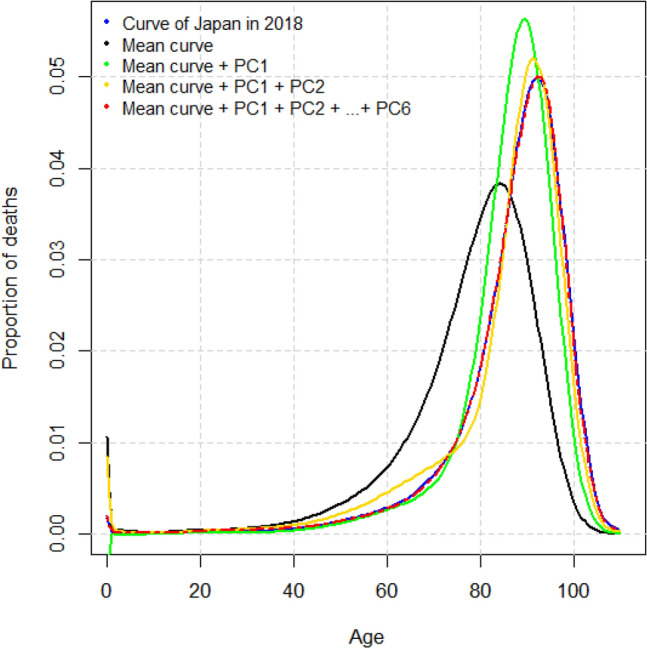
Distance-based clustering for the women’s data: reconstruction of a smoothed curve according to the Karhunen-Loève decomposition property for the choice of the appropriate number of functional principal components

**Fig. 8:**
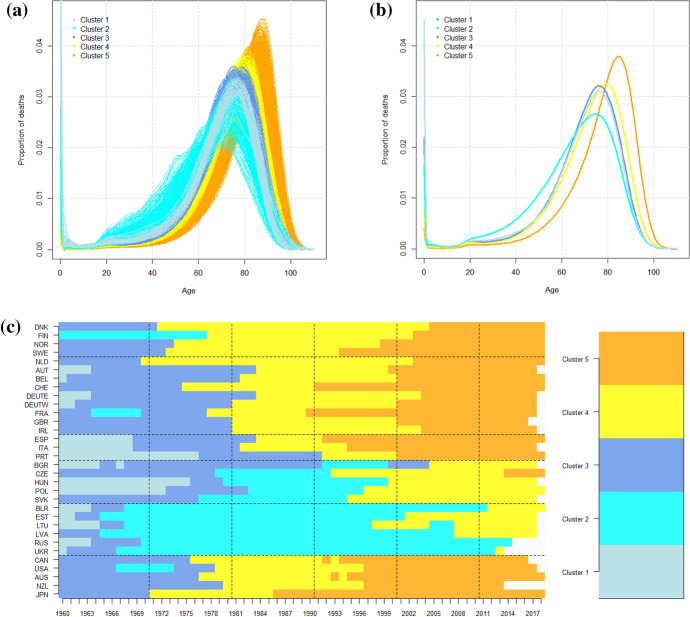
Two-stage clustering for the men’s data: **a** mortality curves, **b** mean curves and **c** composition of the five clusters

**Fig. 9:**
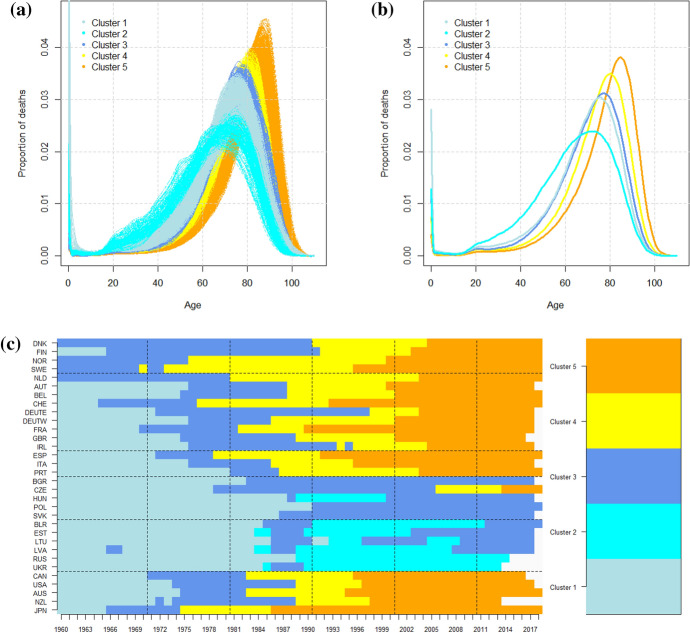
Distance-based clustering for the men’s data: **a** mortality curves, **b** mean curves and **c** composition of the five clusters

**Fig. 10:**
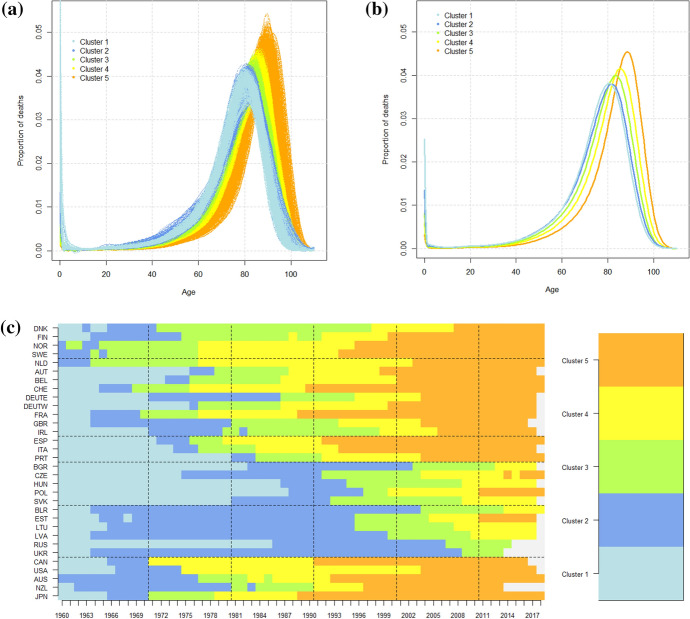
Two-stage clustering for the women’s data: **a** mortality curves, **b** mean curves and **c** composition of the five clusters

**Fig. 11:**
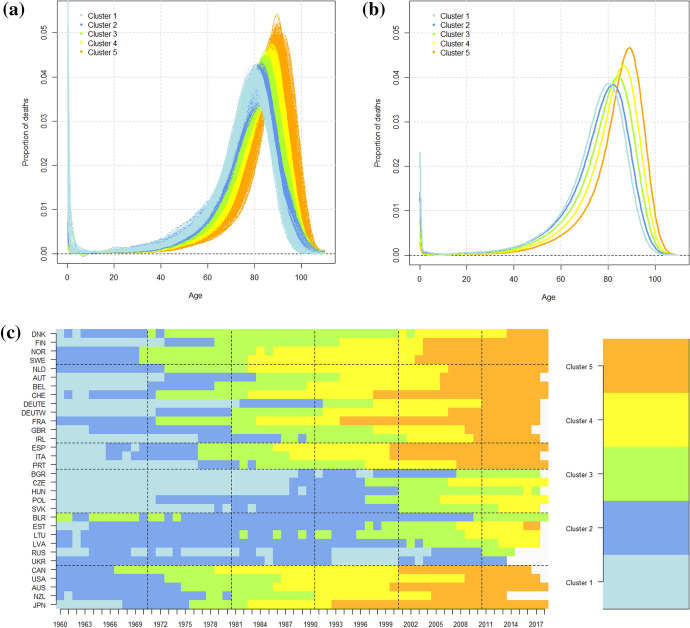
Model-based clustering for the women’s data: **a** mortality curves, **b** mean curves and **c** composition of the five clusters

**Fig. 12:**
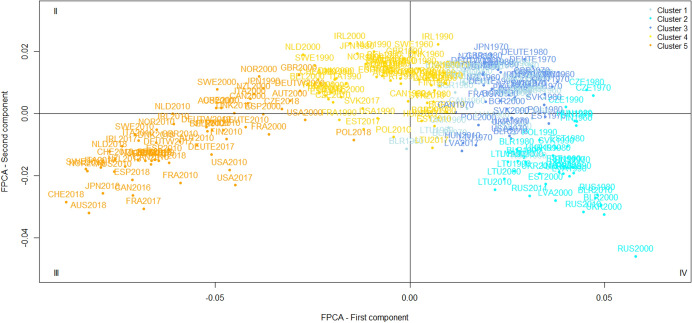
Results of the FPCA for the men’s data—the first principal subspace with all the countries and intervals of 5 years. The colours indicate the group memberships provided by the model-based clustering

**Fig. 13:**
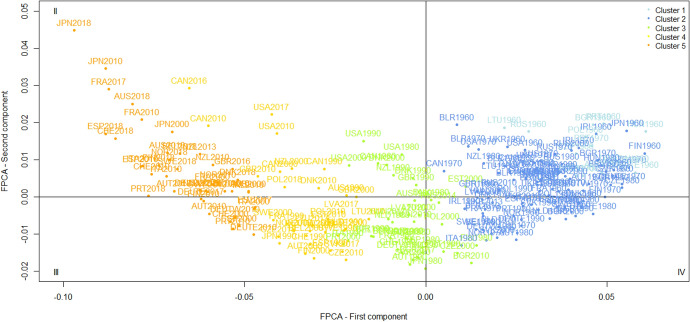
Results of the FPCA for the women’s data—the first principal subspace with all the countries and intervals of 5 years. The colours indicate the group memberships provided by the distance-based clustering

## Data Availability

Data from the Human Mortality Database (downloaded on 29th May 2020) are used in this paper. The full dataset and documentation can be downloaded from https://www.mortality.org/.

## Appendix 1

The decomposition property of Karhunen-Loève turns out to be useful for the evaluation of the appropriate number of principal components to reconstruct the smoothed curves. In Fig. 7, we show the effect of adding the principal components one at a time to the women’s mean curve for Japan in 2010. Clearly, the first six components are needed to obtain the smoothed curve.

## Appendix 2

The other methods of clustering for the men’s and women’s data are presented in this appendix and show similar evolutions based on the same components of mortality. Some differences can be noted for the women’s data with respect to the distance-based method, as the two-stage and model-based cluster analyses emphasised infant mortality. Concerning the men’s data, distance-based clustering identified more precisely the curves with a higher premature mortality; they were restricted to Eastern countries after the 1990s. In addition, two-stage and distance-based clustering of the men’s data highlighted a shift and compression of curves for Baltic countries not detected by the model-based clustering.

See Figs. 8, 9, 10, 11.

## Appendix 3

The figures show that the results obtained for seven selected countries for the men’s and women’s data were confirmed by looking at the principal subspaces with all the countries. The first axis discriminates the majority of countries throughout the whole period from quadrant I to quadrant II; the decrease of the scores reflects the shift of mortality curves toward older ages, although with different timings. The second axis indicates the levels of premature mortality for men.

See Figs. 12 and 13.

## Footnotes

1 The R code used is available on github, so the results are fully replicable.

## Code availability (software application or custom code)

Code for data pre-processing and analysis is available on request from the corresponding author. Codes were executed with R version 3.5.2.
